# Irradiated Human Dermal Fibroblasts Are as Efficient as Mouse Fibroblasts as a Feeder Layer to Improve Human Epidermal Cell Culture Lifespan

**DOI:** 10.3390/ijms14034684

**Published:** 2013-02-26

**Authors:** Francis Bisson, Éloise Rochefort, Amélie Lavoie, Danielle Larouche, Karine Zaniolo, Carolyne Simard-Bisson, Odile Damour, François A. Auger, Sylvain L. Guérin, Lucie Germain

**Affiliations:** 1 Centre LOEX de l'Université Laval and, LOEX/CUO-Recherche, Génie tissulaire et régénération, LOEX—Centre de recherche FRQS du CHU de Québec, Québec, QC G1J 1Z4, Canada; E-Mails: francis.bisson.1@ulaval.ca (F.B.); eloise.r@hotmail.com (É.R.); amelie.lavoie.5@ulaval.ca (A.L.); danielle.larouche.2@ulaval.ca (D.L.); caroline.simard-bisson.1@ulaval.ca (C.S.-B.); francois.auger@fmed.ulaval.ca (F.A.A.); 2 Départements de Chirurgie and d'Ophtalmologie, Faculté de Médecine, Université Laval, Québec, QC K1A 0W9, Canada; E-Mail: karine.zaniolo@gmail.com; 3 Banque de Tissus et Cellules HCL, Laboratoire des Substituts Cutanés (LSC) CNRS UPR-412, Hôpital Edouard Herriot, Lyon 62437 CEDEX03, France; E-Mail: odile.damour@chu-lyon.fr

**Keywords:** feeder layer, i3T3, Sp1, human, skin, keratinocyte, differentiation, proliferation, microarray, gene profiling

## Abstract

A fibroblast feeder layer is currently the best option for large scale expansion of autologous skin keratinocytes that are to be used for the treatment of severely burned patients. In a clinical context, using a human rather than a mouse feeder layer is desirable to reduce the risk of introducing animal antigens and unknown viruses. This study was designed to evaluate if irradiated human fibroblasts can be used in keratinocyte cultures without affecting their morphological and physiological properties. Keratinocytes were grown either with or without a feeder layer in serum-containing medium. Our results showed that keratinocytes grown either on an irradiated human feeder layer or irradiated 3T3 cells (i3T3) can be cultured for a comparable number of passages. The average epithelial cell size and morphology were also similar. On the other hand, keratinocytes grown without a feeder layer showed heavily bloated cells at early passages and stop proliferating after only a few passages. On the molecular aspect, the expression level of the transcription factor Sp1, a useful marker of keratinocytes lifespan, was maintained and stabilized for a high number of passages in keratinocytes grown with feeder layers whereas Sp1 expression dropped quickly without a feeder layer. Furthermore, gene profiling on microarrays identified potential target genes whose expression is differentially regulated in the absence or presence of an i3T3 feeder layer and which may contribute at preserving the growth characteristics of these cells. Irradiated human dermal fibroblasts therefore provide a good human feeder layer for an effective expansion of keratinocytes *in vitro* that are to be used for clinical purposes.

## 1. Introduction

The culture of keratinocytes *in vitro* has many clinical and research applications such as the treatment of chronic ulcers or burn patients, tissue-engineered skin for wound healing or pharmacological studies [1–3]. These epithelial cells, however, rapidly lose their proliferative capabilities and differentiate early when cultured *in vitro* under inappropriate conditions [4,5]. For clinical purpose, the largest amount of skin grafts should be produced from the smallest initial skin biopsy since the surface area of spared sites available from the patient is often limited. It is therefore necessary that culture conditions allow a good proliferation of keratinocytes by delaying their terminal differentiation, as well as maintaining their capacity to act like normal skin epithelial cells once grafted on a patient, *i.e*., ensuring long-term regeneration of the epidermis. In 1975, Rheinwald and Green developed a method to produce cultured epithelial autografts (CEA), consisting of skin epithelial cells cultured with irradiated mouse 3T3 fibroblasts (i3T3) allowing the formation of a thin epithelial sheet suitable for grafting [5]. This technique was rapidly applied clinically for the treatment of severely burned patients [6,7]. However, the molecular effects of the feeder layer on keratinocytes still remain poorly understood.

The use of i3T3 fibroblasts in keratinocyte culture is very common due to their availability. The discovery that the addition of irradiated 3T3 cells limits human fibroblasts proliferation and prevents the invasion of keratinocytes cultures by the fibroblasts has solved a problem that limited the efficient production of epithelial cell sheets [5]. Even if the i3T3 feeder layer system proved to be efficient for keratinocyte expansion *in vitro*, the use of a human feeder layer in a clinical context is, however, suitable as it eliminates the risk of introducing animal components or viruses in the grafted skin sheets [8]. However, very few studies have been conducted using human feeder layers [9–13] and the differences between human and mouse feeder layers have not been extensively investigated.

For cultivation of human keratinocytes, serum-free and/or feeder layer-free culture methods using defined medium have been proposed [14]. These media are useful in a research context because they allow the study of single cell populations as opposed to co-culture systems where the properties of different cell types are more difficult to distinguish from each other. However, in a clinical context, it is of great importance to assess the quality of keratinocytes grown in various conditions before using them for transplantation in order to prevent the tragic loss of the epithelium after grafting [15].

We recently demonstrated that monitoring the expression of general transcription factors such as Sp1 and NFI is of a considerable interest in order to evaluate the quality of keratinocyte cultures prior to their use for the production of reconstructed skin tissues [16,17]. The ubiquitously expressed Sp1 protein is implicated in the regulation of a multitude of cellular processes, notably in the cell cycle [18,19]. Sp1 expression levels have been shown to remain elevated during proliferation of keratinocytes, then decrease as the cells differentiate, and almost disappear when the cells are fully differentiated [17]. The Sp1 expression level thus represents an effective tool to compare the effect of different culture conditions on the proliferative potential of keratinocytes, and adds up to macroscopic analysis when their differentiation state is not easily distinguishable [20].

The aim of this study was to compare the effect of irradiated human dermal fibroblasts feeder layers, as well as feeder layer-free culture methods using different culture media, on keratinocyte growth *in vitro*. For this purpose, keratinocytes were amplified with an i3T3 feeder layer before changing with specific tested conditions. Our results show that a human feeder layer is efficient to both maintain keratinocyte proliferation and delay their massive terminal differentiation. High expression levels of Sp1 are preserved in human feeder layers compared to the tested defined media. Keratinocytes grown with feeder layers reached a high number of passages in culture. These results suggest that human fibroblasts prevent the early terminal differentiation of keratinocytes through a molecular pathway involving Sp1, whereas defined media and the absence of a feeder layer lack the ability to maintain Sp1 expression, which correlates with the loss of the keratinocyte proliferative capability. In addition, gene profiling on microarrays also identified potential target genes other than Sp1 that may also contribute to the maintenance of the growth and morphological properties of skin keratinocytes when they are grown along with an i3T3 feeder layer.

## 2. Results

### 2.1. Cell Morphology and Growth Rate of Keratinocytes Cultured on Human or i3T3 Feeder Layers Are Comparable

Keratinocytes were isolated and cultured with an i3T3 feeder layer for amplification before transferring to each specific tested conditions at passage 4. To compare the impact of a human dermal fibroblast feeder layer with that of an i3T3 feeder layer on cell growth, human keratinocytes were cultured with both feeder layers in complete culture medium (DME-F12). Although keratinocytes are usually cultured up to 3 or 4 passages when used in clinical applications, we subcultured the cells until senescence. Human dermal fibroblasts from two different donors were tested: irradiated human feeder layer-1 and -2 (iHFL1, iHFL2). Human fibroblasts are larger than 3T3, thus a lower number of human cells was used in feeder layers in order to compensate for this variation in size [21]. Moreover, human feeder layers remain stable over several weeks after irradiation [22] whereas 3T3 detach from the flasks after 7–10 days. Both the seeding fibroblast density for each feeder layer and the conditions selected (seeding the 3T3 at the same time than the keratinocytes or seeding iHFL one week before keratinocytes) were adjusted to obtain an optimal equivalent keratinocyte growth rate. This is critical in order to obtain the same number of keratinocytes at confluence (data not shown). In a similar way, to evaluate whether keratinocyte expansion *in vitro*, using defined media developed for serum/feeder layer-free keratinocyte culture systems, is equivalent to feeder layer culture systems, keratinocytes were also cultured without any feeder layer in complete DME-F12 (control), KGM-2, DKSFM or KGM-CD medium. Once keratinocytes have covered an area of 80%–90% (referred to as near-confluence), they were sub-cultured (passage). At each passage, the morphology, size and growth rate of keratinocytes were analyzed.

**Figure 1:**
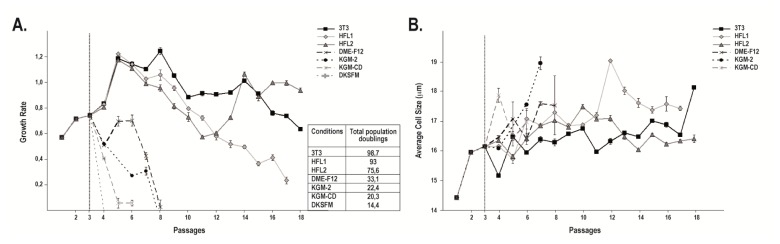
Growth rate and average cell size of keratinocytes over culture passages. (**A**) Growth rate of keratinocytes (Kfl57) calculated as the number of population doubling per day on average. The total population doubling reached by keratinocytes cultured in each condition is indicated in the table. Note that in the HFL1 conditions, keratinocytes terminally differentiated at passage 17 whereas cells in the HFL2 and 3T3 conditions were stopped at passage 18. (**B**) Average cell size in μm. Error bars represent standard error of the mean per passage per condition (*n* = 3). For both analyses, all conditions were amplified until passage 3 with an i3T3 feeder layer. Cells were grown in their respective conditions starting at the 4th subculture as described in Materials and Methods.

**Figure 2:**
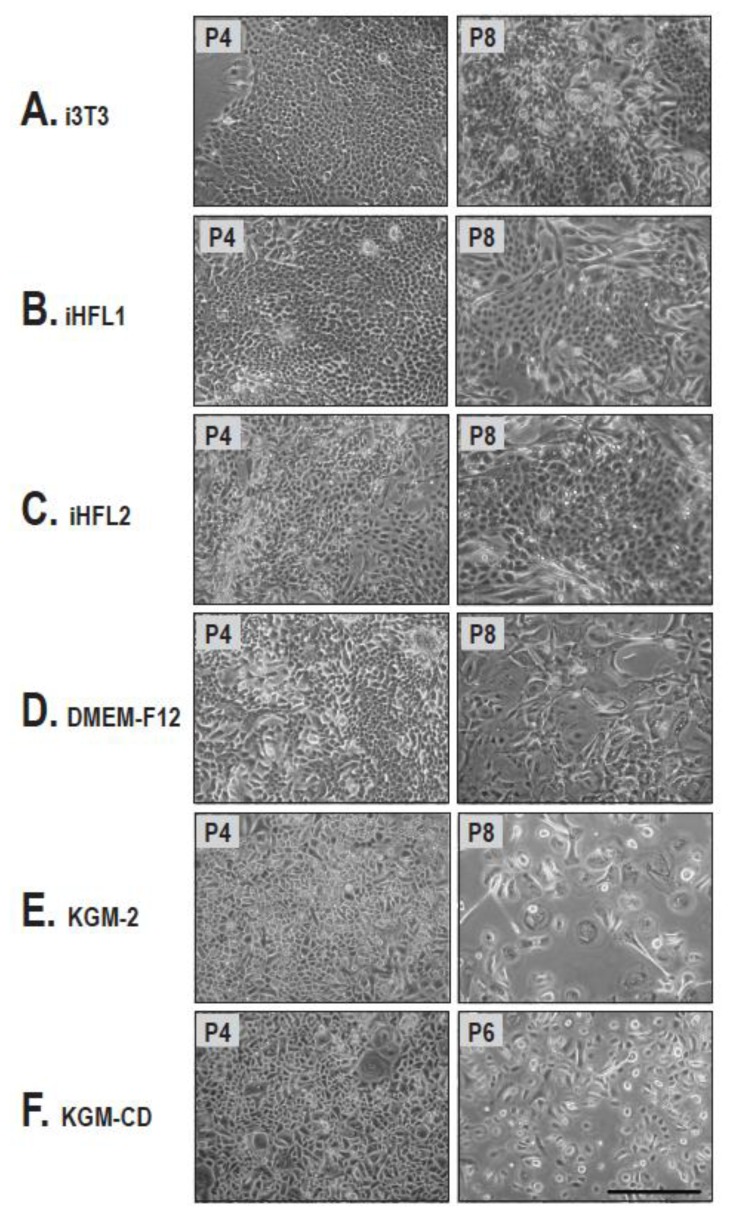
Keratinocytes morphology over culture passages. (**A**–**C**) General morphology of keratinocytes (Kfl57) grown with i3T3 (**A**), iHFL1 (**B**), iHFL2 (**C**) feeder layers or without (**D**–**F**) feeder layer in DME-F12 (**A**–**D**), KGM-2 (**E**) and KGM-CD (**F**) as observed under phase contrast microscopy. Note that at early passages (left column), cultures contained uniformly small keratinocytes with a high nucleus to cytoplasm ratio typical of less differentiated epithelial cells compared with more advanced passages (right column). Note that without feeder layers (**D**–**F**) keratinocytes presenting a differentiated morphology emerged rapidly over passages. Bar = 350 μm.

**Figure 3:**
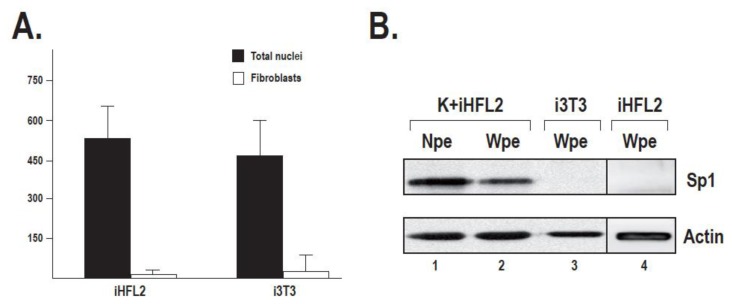
Fibroblast proportion in near confluence keratinocyte cultures and Sp1 expression in fibroblasts. (**A**) Number of vimentin-expressing fibroblasts compared with total nuclei count in keratinocytes cultured with iHFL2 or i3T3. (**B**) Expression of Sp1 was monitored by Western blot on 40 μg nuclear (lane 1, Npe) or whole cell protein extracts (lane 2, Wpe) from keratinocytes grown with iHFL2 (K + iHFL2) at near-confluence, 20 μg whole cell protein extract from i3T3 (lane 3) and 4 μg whole cell protein extract from iHFL2 (lane 4). The 4- and 20-μg protein extracts prepared from either iHFL2 or i3T3 are respectively twice and five times more than that of fibroblast proteins estimated in the 40 μg whole keratinocyte/feeder layer protein extracts. Note that no Sp1 protein is detectable in feeder layer cells, indicating that fibroblasts contribution to the Sp1 expression signal in near-confluence keratinocyte culture extracts is negligible.

**Figure 4:**
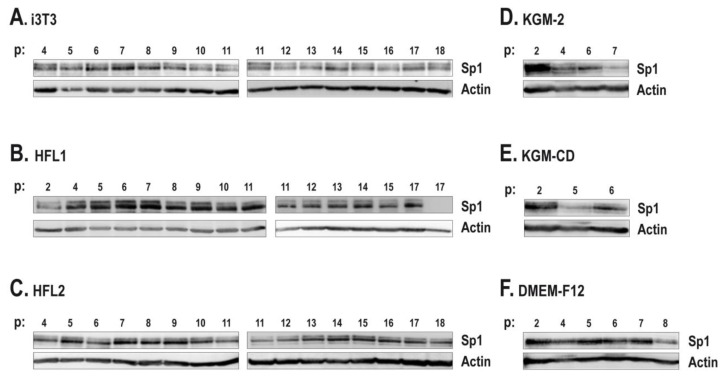
Western blot analysis of Sp1 expression level for each condition and each passage. The level of Sp1 expression was analyzed using whole cell protein extracts (40 μg). Expression levels were analyzed for keratinocytes (Kfl57) cultured with i3T3 (**A**), iHFL1 (**B**) or iHFL2 (**C**) feeder layers, or without feeder layer in KGM-2 (**D**), KGM-CD (**E**) or DME-F12 (**F**). Although the KGM-2 condition (**D**) entered terminal differentiation at passage 8, not enough proteins were collected and therefore no Sp1 expression analysis was conducted at this passage. For every condition, the level of actin expression was monitored for normalization purpose.

**Figure 5:**
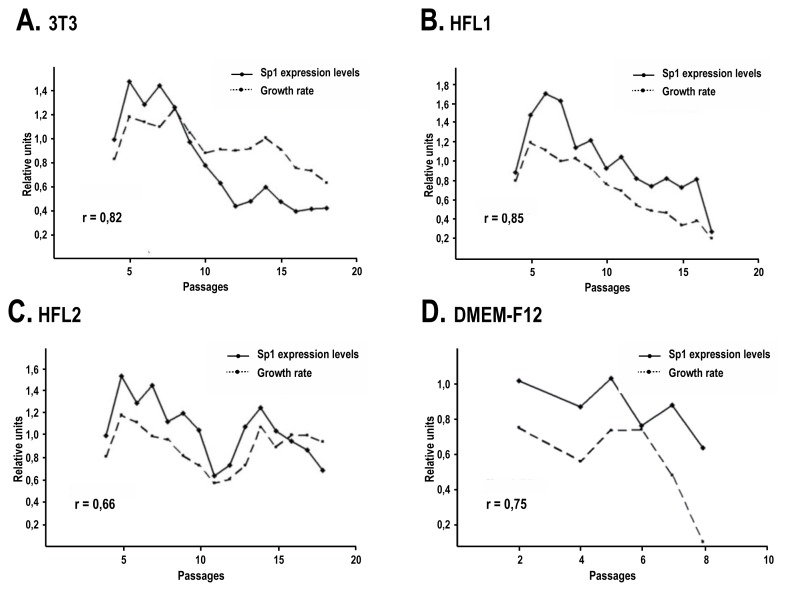
Correlation between cellular growth rate and densitometric analysis of Sp1 expression levels. Densitometric analysis was performed on Western blotted Sp1. Sp1 values were normalized by a densitometric analysis of actin expression levels. Sp1 expression levels are expressed in relative units for comparison purpose. Growth rate data are the same as those presented in Figure 1. “r” represents Pearson’s correlation coefficient.

When grown on either a mouse or human feeder layer, human keratinocytes reached a high number of passages before they became senescent. Indeed, keratinocytes cultured with iHFL1 reached passage 17 before terminally differentiating while keratinocytes cultured with i3T3 and iHFL2 did not reach senescence by passage 18 (Figure 1A). These cultures were used for the following figures (1B-5). Without a feeder layer, the proliferative capacity of keratinocytes was quite limited as they could reach a maximum of 8 passages when grown on DME-F12 and KGM-2 whereas they only reached passage 6 when grown on KGM-CD before they stopped proliferating (Figure 1A). Cells cultured in DKSFM never reached confluence and no data was gathered regarding that condition.

Given that the keratinocyte cell size increases upon terminal differentiation [23], the average cell size was analyzed for the whole population at each passage for every condition. On average, the cell size increased a few passages before the cells underwent their final passage (Figure 1B). One exception was noted when the average size of keratinocytes grown on the first human feeder layer (iHFL1) suddenly increased at passage 12 but stopped growing only 5 passages later, at passage 17.

Cell morphology represents another important biomarker. Indeed, less differentiated keratinocytes are characterized by a smaller size with a high nucleus to cytoplasm ratio while differentiated keratinocytes present a small nucleus to cytoplasm ratio [24,25]. We therefore observed keratinocytes grown using the various culture conditions described above under phase contrast microscopy. No noticeable difference on overall keratinocyte morphology over passages was observed between cells cultured along with either human and mouse feeder layers (Figure 2A–C). As expected, the number of cells presenting a high nucleus to cytoplasm ratio gradually decreased with passage number. On the other hand, cells grown in defined media looked small and polygonal, typical of cells grown at low passages in low-calcium medium [26]. Their progression towards terminal differentiation was recognizable by the gradual appearance of heavily bloated cells (Figure 2E,F). Unlike keratinocytes cultured with mouse or human feeder layers, cells presenting a differentiated morphology emerged rapidly over passages when no feeder layer was used, regardless of culture medium treatments (Figure 2D–F).

Altogether, these results suggest that the impact of a human dermal fibroblast feeder layer on the keratinocytes growth rate, growth arrest, average cell size and overall morphology is equivalent to that observed using mouse i3T3 as a feeder layer. Without a feeder layer, keratinocytes rapidly showed signs of terminal differentiation and could not be expanded as much as keratinocytes cultured with fibroblast feeder layers. For instance, cells cultured with iHFL1 had on average 75.6 ± 0.2 (*n* = 3) population doublings whereas the highest number of population doublings yielded in the absence of a feeder layer was 33.1 ± 1.1 (*n* = 3).

### 2.2. Sp1 Expression in Fibroblasts Used as Feeder Layers Is Negligible

To evaluate whether human dermal feeder layers exert their positive influence on keratinocyte growth through stabilization of the transcription factor Sp1, as we recently observed when keratinocytes are grown along with i3T3 [17], we examined Sp1 expression in protein extracts prepared from each culture condition by Western blotting. In order to correct for the fibroblast contribution to the Sp1 signal in keratinocytes cultured with i3T3 or iHFL2, we determined the proportion of fibroblasts remaining at near-confluence, which is the time point where the protein extracts were collected. The proportion of fibroblasts remaining at the moment keratinocytes were harvested (near confluence) for protein preparation was then quantified by immunofluorescence assays performed on cells cultured on coverslips and using vimentin and keratin 14 as fibroblast and keratinocyte markers, respectively. We calculated that 2.9% ± 3.1% (*n* = 15) of irradiated human fibroblasts and 8.1% ± 10.9% (*n* = 14) of i3T3 remained after keratinocytes reached near-confluence (Figure 3A). The difference can probably be accounted for the different seeding density of each type of feeder layer (8000 cells/cm^2^ for iHFL2 against 20,000 cells/cm^2^ for mouse 3T3). Therefore, to evaluate the fibroblast contribution to the Sp1 signal, a control Western blot experiment was conducted using proteins extracted from fibroblasts cultured alone (Figure 3B). These analyses revealed no expression of Sp1 in iHFL2 nor i3T3 under these conditions (Figure 3B). We therefore assume that the Sp1 signals observed in Figure 3B originated solely from keratinocytes and not from fibroblasts.

### 2.3. The Human Feeder Layer Preserves Sp1 Expression in Keratinocytes

Sp1 expression levels were next analyzed by Western blot using the protein extracts prepared from the above conditions, at each cell passage. Our results suggest that aside from helping keratinocytes to grow for a greater number of passages, i3T3, as well as human feeder layers, helped maintain a basal level of Sp1 expression over a higher number of passages (Figure 4A–C). Sp1 expression fluctuates over passages. Typically, Sp1 expression increased during the early passages (Figure 4A–C) and dropped concomitantly with the appearance of terminal differentiation signs such as morphological changes and a reduction of the growth properties (Figures 1A and 2). Interestingly, a drop in Sp1 expression was observed in the iHFL2 condition that corresponds to the lower growth rate of keratinocytes near passages 11 and 12. Pearson’s correlation coefficient was next determined in order to verify for a possible link between the growth rate variability observed over cell passages with the relative expression of Sp1, as determined by band densitometric analysis. A moderate correlation was observed in the iHFL2 condition (*r* = 0.66) and a high correlation was observed in the other two feeder layer conditions (*r* = 0.85 and 0.82 for iHFL1 and i3T3 respectively) (Figure 5A–C).

In the absence of any feeder layer, Sp1 expression level was noticeably reduced. A quick drop in the expression of Sp1 was observed as early as passage 4 or 5 (Figure 4D,E). This is consistent with their lower growth rate and visible advanced differentiation state (Figures 1A and 2E,F). Unlike when cultured with a feeder layer (Figure 4A–C), keratinocytes cultured in DME-F12 alone had no increase in Sp1 expression following the first passages, the Sp1 level remaining fairly stable over passages (Figure 4F). A quick drop was noted at passage 8 (Figure 4F) that corresponds to the last passage reached by keratinocytes before they terminally differentiated (Figure 1A). Yet, Sp1 expression was again related to the keratinocyte growth rate as a high correlation (*r* = 0.75 for DME-F12, *r* = 0.92 for KGM-2 and *r* = 0.96 for KGM-CD) was noted between growth rate and Sp1 expression (Figure 5D and data not shown).

Altogether, these results suggest that all feeder layers tested, including the two human dermal fibroblasts, exert a positive influence on keratinocyte growth through maintaining Sp1 expression. The correlation between Sp1 expression levels and the growth rate also suggests that a high Sp1 expression level attests of the good proliferative state of keratinocytes.

### 2.4. The Pattern of Genes Expressed by Human Skin Keratinocytes Differs in the Absence or Presence of a Feeder Layer

**Figure 6:**
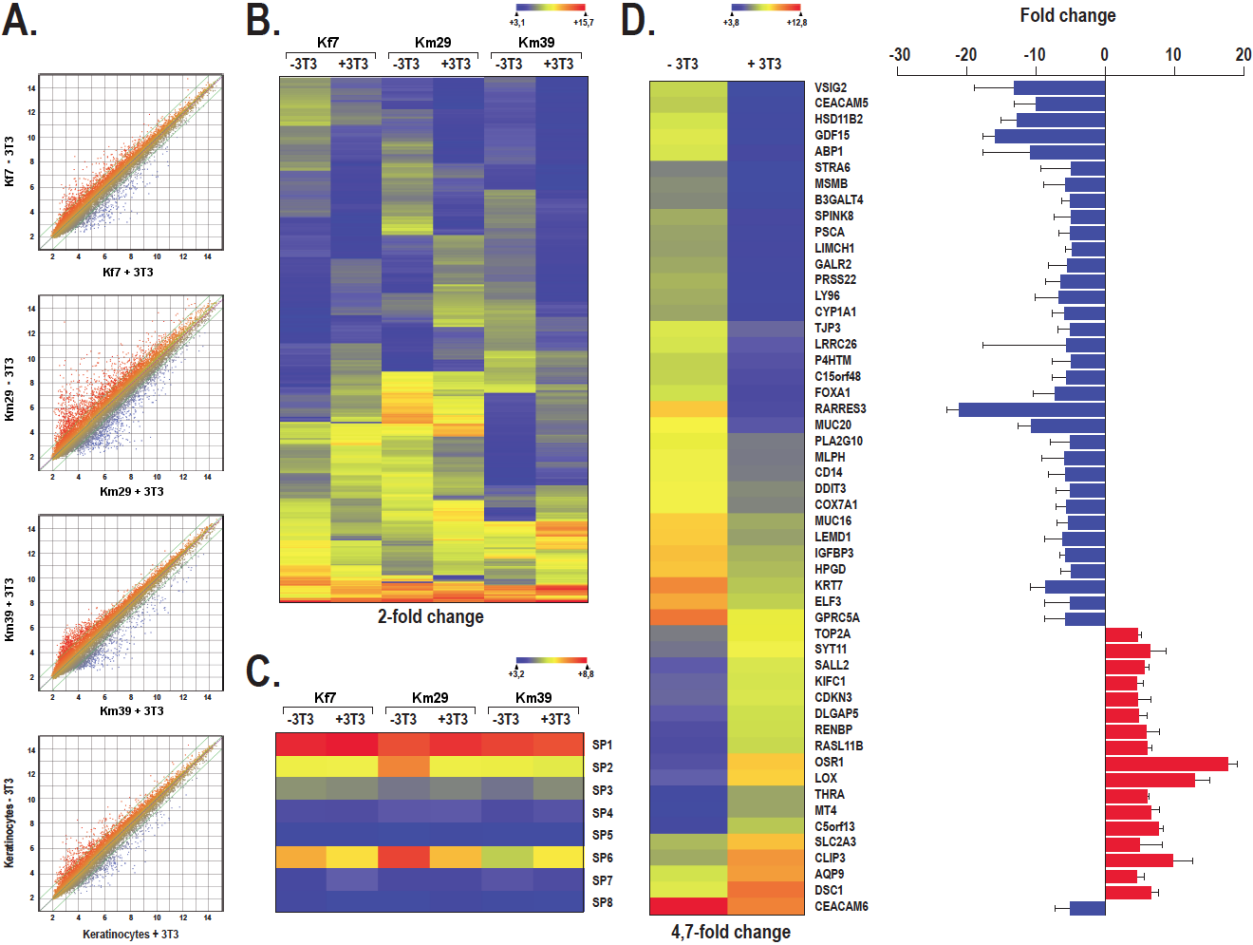
Data from gene expression profiling by microarrays. (**A**) Scatter plots of log_2_ of signal intensity from 48,804 probes for different targets covering the entire human transcriptome of keratinocytes (Kf7, Km29 and Km39) grown with (+3T3; *x*-axis) or without (-3T3; *y*-axis) i3T3. The two-fold change in intensity lines are shown in green. The scatter plot at the bottom of panel A shows the average of the signals yielded by each keratinocyte cell strain grown with (keratinocytes + 3T3) or without (keratinocytes-3T3) a feeder layer. (**B**) Heatmap representation of genes whose expression is differentially regulated by at least 2-fold in keratinocytes grown with a feeder layer (Kf7 + 3T3, Km29 + 3T3 and Km39 + 3T3) against keratinocytes grown alone (Kf7-3T3, Km29-3T3 and Km39-3T3). (**C**) Heat map representation of the transcription factors Sp1 to Sp8 expressed by keratinocytes grown with a feeder layer (Kf7 + 3T3, Km29 + 3T3 and Km39 + 3T3) against keratinocytes grown in the absence of i3T3 (Kf7-3T3, Km29-3T3 and Km39-3T3), as determined by microarrays. (**D**) Left: Heatmap representation of genes whose expression is modified by at least 4.7-fold in keratinocytes grown with a feeder layer (Keratinocytes + 3T3: corresponds to the average of the signals from each individual cell-strain grown with i3T3) against keratinocytes grown alone (keratinocytes-3T3: corresponds to the average of the signals from each individual cell-strain grown without i3T3). Right: graphical representation of the linear fold changes for each of the 52 genes appearing on the heatmap. Blue: repressed genes; red: activated genes.

We next conducted gene expression profiling by microarray analyses on total RNAs isolated from human skin keratinocytes grown with or without i3T3 to sort out genes that are the most differentially regulated in the absence or presence of a feeder layer at the transcriptional level. A scatter plot analysis of the 48,804 transcripts contained on the HumanHT-12 v3 Expression BeadChip arrays indicates clearly that keratinocytes grown in the presence of a feeder layer of i3T3 (Kf7 + 3T3, Km29 + 3T3 and Km39 + 3T3) have patterns of expressed genes very distinctive from those yielded by the same cells grown without a feeder layer (Kf7-3T3, Km29-3T3 and Km39-3T3), as revealed by the dispersion of the normalized signals that appear as a cloud of dots on Figure 6A. Analysis of the linear regression curve for each cell strain (purple lines on Figure 6A) indicates, however, that there are almost as many genes that become repressed than there are that are activated among genes differently regulated in the absence or presence of the feeder layer.

In an attempt to visualize the variation between keratinocytes grown with or without i3T3, a heatmap was generated for all the genes showing a 2-fold or more expression variation unique to each cell strain grown with i3T3 paired against the expression profile of their corresponding cell strain grown without i3T3. A total of 675- (for Kf7), 1791- (for Km29) and 862 genes (for Km39), corresponding to 1.4%, 3.7% and 1.8% of all the targets contained on the array, respectively, fitted into that category of differentially regulated genes (Figure 6B). Clustering of the *in vivo* microarray data for all the genes from the Sp1 sub-family expressed in keratinocytes grown with or without i3T3 into a heatmap indicated clearly that there is no significant variation in the expression of these genes, including Sp1 (Figure 6C), a result consistent with those recently reported by our laboratory [16]. The filters from the Arraystar software were then set to select genes with at least a 4.7-fold change in expression (activated or repressed) between keratinocytes grown with or without i3T3. This search identified 52 genes that fit this criteria, of which 35 were repressed and 17 activated in the presence of a feeder layer (Figure 6D,E and Table S1).

## 3. Discussion

In a clinical context, cultured epithelial cells must have the ability to ensure the long-term regeneration of the epithelium once grafted. Human skin epithelial cells can be grown in culture in the presence of a feeder layer of irradiated fibroblasts (such as i3T3) and medium supplemented with various additives that prolong lifespan and increase the growth rate of keratinocyte cultures *in vitro*[5,27–29]. Furthermore, the presence of a feeder layer in keratinocyte cultures does not generate transformed cells and is not associated with an increased risk of tumorigenicity [30]. Fibroblasts feeder layers also allowed the culture of many different types of epithelial cells such as those from the corneal epithelium [31], the oral epithelium [32], the bronchial epithelial cells [33] as well as pathological skin epithelial cells [34]. In the present study, we confirmed the clear advantage conferred by feeder layers on large scale expansion of keratinocytes *in vitro* compared to serum/feeder layer free systems. In accordance with the study of Auxenfans and colleagues showing that stem cells are preserved as well, if not better, with irradiated human feeder layers compared with i3T3 [9], our results further support the fact that selected irradiated human dermal fibroblasts are an appropriate replacement for i3T3 as feeder layers in a clinical context. Furthermore, we demonstrated that properties of cultured keratinocyte populations, such as the maximum number of passages they can reach in culture, the average cell size at each passage, the general morphology as well as the level of Sp1 expression, which is an indicator of good proliferation potential for keratinocytes [17,20], were equivalent when grown on either a human dermal fibroblasts or i3T3 feeder layer. This is an important optimization step when the goal consists in using these cultured cells for clinical applications.

As previously reported [17,20], some variations in the level of Sp1 expression were observed in epithelial cell cultures throughout the passages. Typically, Sp1 expression increased in the first passages, and then progressively decreased, before it reached very low levels, concomitantly with the appearance of terminal differentiation signs such as morphological changes and a lower growth rate. Interestingly, the Sp1 variations we observed followed the growth rate of the corresponding keratinocyte populations. This is consistent with the function played by Sp1 in the cell cycle progression [18]. One good example is the decrease in the level of Sp1 expression at passages 11 and 12 observed with keratinocytes grown along with iHFL2 that perfectly matches with a corresponding reduction in the growth rate of these cells and a rise in their average cell size. Although these changes in the expression of Sp1 at the protein level are clearly significant, they apparently did not result from corresponding changes in the transcription of this gene. Indeed, gene profiling on microarrays revealed no significant change in the transcription of the Sp1 gene when keratinocytes are grown with a feeder layer (see Figure 6C). However, this result is consistent with our recent finding that human skin keratinocytes maintain a higher amount of Sp1 at the protein level (and also Sp3 and NFI) when grown in the presence of a feeder layer, not through a corresponding change in the transcription of that gene but rather by preventing Sp1 from being degraded by endogenous proteases [16]. Sp1 has been reported as being specifically degraded by a yet unidentified, trypsin-like serine protease in the rat lung and HL60 cells [35,36]. Interestingly, among the genes differentially regulated by the feeder layer, PRSS22/tryptase ɛ, which encodes for a self-activated serine protease [37], turned out to be expressed to high levels in the epithelial cells from the skin [38]. Data from the microarrays indicate that transcription of the PRSS22/tryptase ɛ gene is almost entirely repressed in keratinocytes grown in the presence of i3T3. Besides PRSS22/tryptase ɛ, other serine proteases also seek their expression reduced in skin keratinocytes grown in the presence of a feeder layer, including TMPRSS4 and most of all, PRSS27/marapsin that has been reported to be highly expressed in squamous epithelia [39] (also refer to Supplementary Figure 1). This could be because the proportion of differentiated keratinocytes is lower in keratinocytes cultured in the presence of a feeder layer.

The growth rate of keratinocytes grown along with iHFL2 was reduced as their average cell size increased at passages 11 and 12. Yet, the reason why the growth rate of keratinocytes started to rise while the average cell size decreases soon after still remains unknown. It is likely that a few remaining stem cells present in the culture began growing to regenerate the entire cell culture. The feeder layer might have played a role in stabilizing the proliferation of these keratinocytes either through an indirect action on Sp1 or by preserving a proportion of the stem cell population, two mechanisms that otherwise would not have been possible in the absence of a feeder layer. The preservation of stem cells *in vitro* was demonstrated by the identification of label-retaining cells in tissue-engineered skin [40]. One other hypothesis would be that the feeder layer somehow modified the expression of genes other than Sp1 that contribute to the growth and morphological properties of keratinocytes compared to keratinocytes cultured without feeder layers. Gene profiling on microarrays identified 52 genes that are regulated differentially by at least 4.7-fold when keratinocytes are grown in the presence of i3T3 compared to those cultured without i3T3. Interestingly, the gene whose transcription is the most differentially regulated by the feeder layer (Retinoic acid receptor responder protein 3 (RARRES3/*TIG3*): 21-fold repression) turned out also to be a well known retinoic acid inducible tumor suppressor gene [41], a gene also involved in growth regulation and epithelial differentiation [42,43]. Similarly, the protein product encoded by GPRC5A, another retinoic acid responding gene whose expression is heavily downregulated by the feeder layer (see Figure 6 and Table S1), was also reported to function as a potential tumor suppressor gene [44,45]. Consistent with the improved properties of keratinocytes grown with a feeder layer, suppression of PRC5A expression in PRC5A(−/−) knockout mice is also associated with increased epithelial lung cell proliferation, resistance to cell death in suspension, and increased basal, tumor necrosis factor alpha-induced, and lipopolysaccharide-induced NF-kappaB activation [46]. However, these authors did not examine the influence that suppression of PRC5A expression might have on the properties of skin epithelial cells. Yet, the possibility remains that the improved growth ability of our strains of human skin keratinocytes, when they are grown in the presence of i3T3, may in part rely on the reduced expression of both these tumor suppressor/growth regulatory genes, thereby delaying their terminal differentiation. These results are consistent with the role of retinoic acid on the regulation of growth/terminal differentiation in keratinocytes [47]. Expression of the gene encoding desmocollin 1 (DSC1) that belongs to one of two classes of desmosomal cadherins that are present in desmosomal junctions typical of epithelia and certain other tissues increased several times (7-fold increase) in response to the presence of a feeder layer. Interestingly, the product of the DSC1 gene has been shown to be intimately related with the keratinization of the epithelial tissues during mouse development [48] and its deficiency also correlates with epidermal fragility in mice [49]. DSC1 is the predominant DSC isoform synthesized in terminally differentiating keratinocytes of stratified epithelia [48,50]. This variation in the expression of DSC1 between the two culture methods is consistent with the excellent desmosome formation when keratinocytes are grown in the presence of feeder layers in a medium containing normal calcium concentration. In addition, six out of the 52 differentially regulated genes encode transcription factors. FOXA1, ELF3 and DDIT3 have their expression increased 5-, 5- and 7-fold, respectively, in the absence of a feeder layer whereas that of SALL2, THRA and OSR1 decreased by 6-, 6- and 18-fold, respectively. However, the true participation of these transcription factors to the improved growth and morphological properties of human skin keratinocytes when they are grown with a feeder layer has yet to be demonstrated.

Besides the above observations, the ultimate testing of cultured tissues remains, however, their functional quality after their transplantation *in vivo*. In the case of skin, where the epithelium is in constant renewal, a good substitute must ensure barrier function and preserve its regenerative properties for the entire patient’s lifespan, a quality reflected by the stem cell components. On this particular aspect, the strong increase in desmocollin-1 (DSC1) gene expression that we observed in keratinocytes grown with an i3T3 feeder layer by microarray analyses may strongly contribute at ensuring a better barrier function as mice deficient in DSC1 expression also have impaired epidermal barrier maintenance function [49]. It is also essential to the formation of cultured autologous epidermal sheets since desmosomes are required for the cohesion between keratinocytes and the formation of a large cohesive tissue instead of single cells. The loss of the epithelium a couple of weeks after grafting of an autologous three-cellular cultured skin substitutes based on esterified hyaluronic acid scaffold has been reported [15]. The degradation of the epithelial layer could be attributed to the loss of stem cells during the culture steps leading to the production of the skin substitute or to an inappropriate stem cell niche within this substitute. In addition, an insufficient quantity of stem cells in the initial material has been associated with graft failure in a minority of patients grafted with cultured corneal epithelia [51]. Although some concerns have been raised regarding the use of feeder layers or animal serum during culture of cells for intended clinical use, our data demonstrate that *in vitro* expansion of human keratinocytes is significantly higher when a fibroblast feeder layer and serum are used compared to all the tested defined culture media proposed for feeder layer-free culture systems. The importance of cell-extracellular matrix and cell-cell contact has been demonstrated [27,52]. Moreover, stem cells are preserved in keratinocytes cultured with feeder layers as demonstrated by tissue-engineered skin substitutes produced from these cultures [40].

The accessibility of skin biopsies and the possibility to harvest and expand epithelial stem cells *in vitro* allow for the production of autologous tissues suitable for grafting. Cultured epithelial autologous grafts (CEA) are usually produced from keratinocytes at low passages (1 to 4). Our study supports the use of human feeder layers as an alternative for the use of mouse i3T3 in keratinocyte cultures. There is a possibility that some allogenic fibroblasts can be transferred among autologous cells. As of now, when using this type of culture conditions, considerations must be implemented; fibroblast banks used for the production of feeder layers must be tested for human viruses, bacterial endotoxins, fungals and mycoplasma contaminations while serum must come from a certified source. In the future, however, an ideal solution for keratinocyte cultures that are to be used for clinical purposes would be a defined medium that allows the creation of fully autologous grafts with the same quality and yield as those produced under culture conditions combining serum and fibroblast feeder layers. In the process of creating such a medium, the understanding of interactions between keratinocytes and the feeder layer, which are currently poorly understood, is an important step. The work presented here highlights a regulation system signaling through the transcription factor Sp1. We hope this knowledge will eventually allow the creation of high quality skin grafts under highly controlled and defined conditions. In the mean time, our results support the notion that the i3T3 feeder layer can be efficiently replaced by selected irradiated human dermal fibroblasts.

## 4. Experimental Section

### 4.1. Cell Culture

This study was approved by the “comité d’éthique de la recherche du Centre hospitalier affilié universitaire de Québec”. Human skin keratinocytes and/or fibroblasts were obtained from a normal newborn or adult (7 days (kF7), 10 days (HFL1) and 4 years old (HFL2) (foreskin), 29 (Km29) and 39 years old (Km39) (mammary) and 57 years old (face-lift (Kfl57) years old) skin specimen removed during surgery of donors after informed consent were given. Cells from different donors were not pooled. Each cell strain was studied separately. Two irradiated human feeder layers were tested; the first (iHFL1) was obtained from the dermis of a human foreskin from a 4 years-old donor as described [27] while the other (iHFL2) was from a 10 day-old donor as described [52]. Upon isolation as previously described [53], keratinocytes were cultured in the presence of an irradiated mouse Swiss 3T3 (i3T3) fibroblast feeder layer (20,000 i3T3 per cm^2^ irradiated at 6000 rad) to prevent further proliferation. The keratinocyte culture medium (referred to as DME-F12) consisted of 3:1 DME (Dulbecco’s modified Eagle’s medium, Invitrogen, Oakville, ON, Canada):Ham (Ham’s F12 medium, Invitrogen) supplemented with 5% Fetal clone II serum (HyClone, Logan, UT, USA), 10 ng/mL epidermal growth factor (EGF; Austral Biologicals, San Ramon, CA, USA), 24.3 μg/mL adenine (Sigma Chemicals, St. Louis, MO, USA), 5 μg/mL insulin (Sigma Chemicals), 0.4 μg/mL hydrocortisone (Calbiochem, La Jolla, CA, USA), 0.212 μg/mL isoproterenol (Sandoz Canada, Boucherville, QC, Canada), 100 IU/mL penicillin G (Sigma Chemicals) and 25 μg/mL gentamicin (Sigma Chemicals), as described [17,28] except for isoproterenol that replaced cholera toxin after thawing). Keratinocytes were frozen and thawed as described [17,28]. For all experiments, keratinocytes were cultured with i3T3 for the first 3 passages in order to expand them and generate enough cells for the following experiments. For the 4th subculture, keratinocytes were plated in triplicate in their respective condition, with the i3T3 feeder layer, or either the irradiated (6000 rad) iHFL1 or iHFL2 in DME-F12 medium, or without feeder layer in DME-F12, KGM-2 (Keratinocyte growth medium-2, Clonetics Biowhittaker, Walkersville, MD, USA), KGM-CD (Keratinocyte growth medium-chemically defined, Clonetics Biowhittaker) or DKSFM (Defined keratinocyte serum free medium, Invitrogen). The i3T3 cells were seeded (20,000 per cm^2^) at the same time as keratinocytes. Irradiated HFL were pre-seeded in flasks at a concentration of 10,000 per cm^2^ for iHFL1 and 8000 per cm^2^ for iHFL2 at least one week prior to being used and were kept in culture for a maximum of one month. Keratinocytes in every condition were passaged when they reached 80%–90% confluence (referred to as near-confluence). Trypsin inhibitor aprotinin (Sigma-Aldrich, Oakville, ON, Canada) was used to stop the enzymatic activity of trypsin in media containing no serum. Keratinocytes were maintained in culture and passaged in their respective medium until they stop proliferating and became senescent (unable to reach confluence) or when they reached passage 18. Cells were observed under an Olympus CKX41 microscope equipped with an Olympus E-620 camera. At each passage, cell number, and the average cell size were analyzed with a Beckman Coulter. Growth rates were calculated using the following formula:

(1) $$\frac{\text{log }((\text{number of cellsobtianed})/(\text{number of cells seeded}))}{\text{log }(2)}$$

### 4.2. Immunofluorescence Analysis

Indirect immunofluorescence labeling was performed on cells grown to approximately 90% confluence on microscope coverslip glasses and then fixed with ethanol (10 min, −20 °C). The coverslips were carefully washed with phosphate-buffered saline (PBS) then incubated with a blocking buffer (1% bovine serum albumin (BSA) in PBS) for 1 h. After PBS rinses, the cells were incubated with the mouse monoclonal anti-vimentin primary antibody (clone 3D1, gift from Dr. Normand Marceau, Centre de Recherche en Cancérologie de l’Université Laval) for 1 h, then washed in PBS and incubated with a fluorescein-5-isothiocyanate (FITC)-conjugated goat anti-mouse antibody (Chemicon International, Temecula, CA, USA) for 45 min. This step and all subsequent incubation steps were performed in the dark. Next, cells were rinsed in PBS and incubated for 1 h with the rabbit monoclonal anti-K14 antibody (Sigma-Aldrich) for 1 h, washed in PBS and incubated with an Alexa 594-conjugated chicken anti-rabbit antibody (Invitrogen Molecular Probes, Oakville, ON, Canada) for 45 min. After PBS washing, cells were stained with Hoechst 33258 to visualize nuclei. Fluorescence was observed under a Nikon Eclipse E600 microscope equipped with epifluorescence, and pictures taken with a Sensys digital camera.

### 4.3. Western Blot

Whole cell protein extracts were homogenized in lysis buffer containing 1% (*v*/*v*) Triton X-100, 0.1% SDS (sodium dodecyl-sulfate), 50 mM Tris (pH 7.4), 150 mM NaCl, 1 mM Ethylenediaminetetraacetic acid, 1 mM sodium orthovanadate and a cocktail of protease inhibitors (Complete, Roche Molecular Biochemicals, Laval, QC, Canada) as previously described [17]. Crude nuclear extracts were prepared as described [54]. Protein extracts were kept frozen at −80 °C until needed, and the protein concentration of each extract was determined by microBCA protein assay kit (Pierce Biotechnology, Rockford, IL, USA). Protein extracts were fractionated by SDS-PAGE (sodium dodecyl sulfate-polyacrylamide gel electrophoresis, 8% polyacrylamide) (40 μg of total protein extracts was loaded unless stated otherwise) and transferred onto nitrocellulose membranes for Western blotting with the following primary antibodies: rabbit polyclonal anti-Sp1 (PEP 2, Santa Cruz Biotechnology, Santa Cruz, CA, USA) or anti-actin (C4, Cedarlane Laboratories Limited, Burlington, ON, Canada). Membranes were then incubated with peroxidase-conjugated goat anti-mouse (Chemicon International) or goat anti-rabbit (Sigma-Aldrich) secondary antibodies. Immunoreactive complexes were visualized using enhanced chemiluminescence (ECL Plus Western blotting detection system, GE Healthcare Bio-Sciences Inc., Baie d'Urfé, QC, Canada) and the Fusion FX7 (Vilber-Lourmat, Marne-la-Vallée, France). Densitometric analysis was performed using Bio1D software (Vilber-Lourmat) and Pearson’s correlation coefficient (*r*) was calculated using Microsoft Excel software. In order to assure that the Sp1 signal observed in keratinocytes cultured with feeder layers was only originating from keratinocytes and not from the feeder cell layer, 4- and 20-μg protein extracts prepared from either iHFL2 or i3T3, respectively, were analyzed for Sp1 expression. Considering the percentage of fibroblasts remaining in the keratinocyte cultures (see results), these amounts of proteins are respectively twice and five times more than that of fibroblast proteins calculated in the 40 μg whole keratinocyte/feeder layer protein extracts, typically used to analyze Sp1 expression level in keratinocyte cultures.

### 4.4. Gene Expression Profiling

Total RNA was isolated from human skin keratinocytes grown to passage 4 in DME-F12 (80%–90% confluence) either alone or in the presence of i3T3 using the RNeasy Mini Kit (QIAGEN, Toronto, ON, Canada). Total RNA was obtained from 3 different preparations of keratinocytes (Kf7Kprep, Km29 and Km39) cultured from 3 different donors (7-day newborn, 29- and 39-years old) on either plastic or with i3T3. Biotinylated cRNA targets were prepared from 150 ng of total RNA using the Illumina TotalPrep RNA Amplification Kit (Ambion, Austin, TX, USA). Then 1.5 μg cRNA was incubated on a HumanHT-12 v3 Expression BeadChip array (48,804 probes, Illumina, San Diego, CA, USA). Slides were then washed, stained and scanned on an Illumina BeadStation 500 according to the manufacturer’s instructions. Data were finally analyzed using the ArrayStar V4.1 (DNASTAR, Madison, WI, USA) software for scatter plots and generation of the heat maps of selected genes of interest. All data generated from the arrays were also analyzed by RMA (“*Robust Multiarray Analysis*”) for background correction of the raw values. They were then transformed in Log2 base and quantile normalized before a linear model was fit to the normalized data to obtain an expression measure for each probe set on each array.

## 5. Conclusions

In conclusion, the best culture conditions for skin epithelial cells require the presence of a feeder layer. The i3T3 feeder layer can be efficiently replaced by selected irradiated human dermal fibroblasts. Patients suffering from skin loss over large surface area can benefit from grafting of these autologous cultured epithelial cells expanded *in vitro* from a small skin biopsy.

## Supplementary Information
