# Supplementary material for: Irradiated Human Dermal Fibroblasts Are as Efficient as Mouse Fibroblasts as a Feeder Layer to Improve Human Epidermal Cell Culture Lifespan
Source: Int J Mol Sci. 2013 Feb 26;14(3):4684–704. doi: 10.3390/ijms14034684 (PMC3634426; doi:10.3390/ijms14034684)
Supplement: Supplementary file 1 [file ijms-14-04684-s001.docx]

Supplementary Information

**Figure S1.** Serin Proteases Whose Expression is modifyed by the Feeder Layer. Heat map representation of the serine proteases TMPRSS4, SPINK8, PRSS@@, TMPRSS2 and PRSS27 expressed by keratinocytes grown with (Keratinocytes+3T3) or without i3T3 (Keratinocytes-3T3), as determined by microarrays.


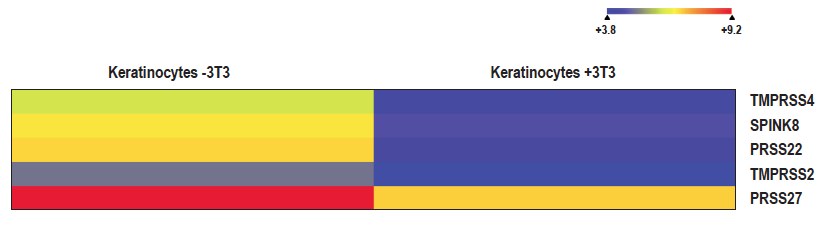


**Table S1.** Biological functions of the protein products encoded by the genes differentially expressed in keratinocytes grown with or without a feeder layer.

| **Gene Name** | **Protein Name** | **Normalized Signal** | | **Fold Change** | **Function** |
| --- | --- | --- | --- | --- | --- |
|  |  | **−3T3** | **+3T3** |  |  |
| **RARRES3** | Retinoic acid receptor responder protein 3 | 628 | 29 | 21 down | Act as a tumor suppressor or growth regulator. |
| **GDF15** | Growth/differentiation factor 15 | 263 | 16,428 | 16 down | Role in regulating inflammatory and apoptotic pathways in injured tissues and during disease processes. |
| **VSIG2** | V-set and immunoglobulin domain-containing protein 2 | 179 | 13 | 13 down | Found in diverse protein families, including immunoglobulin light and heavy chains; in several T-cell receptors such as CD2 (Cluster of Differentiation 2), CD4, CD80, and CD86; in myelin membrane adhesion molecules; in junction adhesion molecules (JAM); in tyrosine-protein kinase receptors; and in the programmed cell death protein 1 (PD1). |
| **HSD11B2** | Corticosteroid  11-beta-dehydrogenase isozyme 2 | 217 | 17 | 13 down | Oxidizes the glucocorticoid cortisol to the inactive metabolite cortisone, thus preventing illicit activation of the mineralocorticoid receptor.A genetically inherited deficiency of HSD11B2 is the underlying cause of the syndrome of apparent mineralocorticoid excess. |
| **ABP1** | Amiloride-sensitive amine oxidase | 230 | 21 | 11 down | Role in controlling the level of histamine and/or putrescine in these tissues. It also binds to and is inhibited by amiloride, a diuretic that acts by closing epithelial sodium ion channels. |
| **MUC20** | Mucin-20 | 406 | 38 | 11 down | May regulate MET signaling cascade. Seems to decrease hepatocyte growth factor (HGF)-induced transient MAPK activation. Blocks GRB2 recruitment to MET thus suppressing the GRB2-RAS pathway. Inhibits  HGF-induced proliferationof MMP1 and MMP9 expression. |
| **CEACAM5** | Carcinoembryonic antigen-related cell adhesion molecule 5 | 164 | 16 | 10 down | Cluster of differentiation 66e. Cell surface glycoprotein that plays a role in cell adhesion and in  intracellular signaling. |

**Table S1.** *Cont.*

| **Gene Name** | **Protein Name** | **Normalized Signal** | | **Fold Change** | **FUNCTION** |
| --- | --- | --- | --- | --- | --- |
|  |  | **−3T3** | **+3T3** |  |  |
| **KRT7** | Keratin, type II cytoskeletal 7 | 1298 | 151 | 9 down | Expressed during differentiation of simple and stratified epithelial tissues. Blocks interferon-dependent interphase and stimulates DNA synthesis in cells. Involved in the translational regulation of the human papillomavirus type 16 E7 mRNA (HPV16 E7). |
| **FOXA1** | Hepatocyte nuclear factor 3-alpha | 200 | 28 | 7 down | Transcriptional activators for liver-specific transcripts such as albumin and transthyretin, and they also interact with chromatin. Similar family members in mice have roles in the regulation of metabolism and in the differentiation |
| **LY96** | Lymphocyte antigen 96 | 116 | 17 | 7 down | Cooperates with TLR4 in the innate immune response to bacterial lipopolysaccharide (LPS), and with TLR2 inthe response to cell wall components from Gram-positive and Gram-negative bacteria. Enhances  TLR4-dependent activation of NF-kappa-B. Cells expressing both MD2 and TLR4, but not TLR4 alone, respond to LPS. |
| **PRSS22** | Brain-specific serine protease 4 | 124 | 19 | 6 down | New member of the chromosome 16p13.3 family of human serine proteases expressed in airway epithelial cells. |
| **LEMD1** | LEM domain-containing protein 1 | 6023 | 98 | 6 down | Among the genes that were frequently transactivated in colorectal tumors, we identified a novel gene termed LEMD1 (LEM domain-containing 1) whose expression was elevated a novel gene termed LEMD1  (LEM domain-containing 1) whose expression was elevated. |
| **CYP1A1** | Cytochrome P450 1A1 | 106 | 18 | 6 down | The cytochrome P450 proteins are monooxygenases which catalyze many reactions involved in drug metabolism and synthesis of cholesterol, steroids and other lipids. This protein localizes to the endoplasmic reticulum and its expression is induced by some polycyclic aromatic hydrocarbons (PAHs), some of which are found in cigarette smoke. The gene has been associated with lung cancer risk. |
| **MLPH** | Melanophilin | 364 | 62 | 6 down | Rab effector protein involved in melanosome transport. Serves as link between melanosome-bound RAB27A and the motor protein MYO5A. A mutation in this gene results in Griscelli syndrome type 3, which is characterized by a silver-gray hair color and abnormal pigment distribution in the hair shaft. |
| **MSMB** | Beta-microseminoprotein | 79 | 14 | 6 down | Member of the immunoglobulin binding factor family. It is synthesized by the epithelial cells of the prostate gland and secreted into the seminal plasma. Role as an autocrine paracrine factor in uterine, breast and other female reproductive tissues. |

**Table S1.** *Cont.*

| **Gene Name** | **Protein Name** | **Normalized Signal** | | **Fold Change** | **FUNCTION** |
| --- | --- | --- | --- | --- | --- |
|  |  | **−3T3** | **+3T3** |  |  |
| **CD14** | Monocyte differentiation antigen CD14 | 361 | 64 | 6 down | Acts as a co-receptor (along with the Toll-like receptor TLR 4 and MD-2) for the detection of bacterial lipopolysaccharide (LPS). |
| **GPRC5A** | Retinoic acid-induced protein 3 | 1757 | 313 | 6 down | Role in embryonic development and epithelial cell differentiation. Involved in interaction between retinoid acid and G protein signalling pathways. Retinoic acid plays a critical role in development, cellular growth, and differentiation. This gene may play a role in embryonic development and epithelial cell differentiation. |
| **IGFBP3** | Insulin-like growth  factor-binding  protein 3 | 683 | 122 | 6 down | Role in regulation of cell growth. IGF-binding proteins prolong the half-life of the IGFs and have been shown to either inhibit or stimulate the growth promoting effects of the IGFs on cell culture. They alter the interaction of IGFs with their cell surfacereceptors. |
| **C15ORF48** | Normal mucosa of esophagus-specific gene 1 protein | 175 | 31 | 6 down | A novel gene, NMES1, downregulated in human esophageal squamous cell carcinoma. |
| **LRRC26** | Leucine-rich  repeat-containing protein 26 | 221 | 40 | 6 down | Auxiliary protein of the large-conductance, voltage and calcium-activated potassium channel (BK alpha). Required for the conversion of BK alpha channels from a high-voltage to a low-voltage activated channel type in non-excitable cells. These are haracterized by negative membrane voltages and constant low levels of calcium characterized by negative membrane voltages and constant low levels of calcium. |
| **GALR2** | Galanin receptor  type 2 | 110 | 20 | 6 down | Important neuromodulator. Receptor for the hormone galanin and for GALP. The activity of this receptor is mediated by G proteins that activate the phospholipase C/protein kinase C pathway (via G(q)) and that inhibit adenylyl cyclase (via G(i)). |
| **COX7A1** | Cytochrome c oxidase subunit 7A1, mitochondrial | 390 | 72 | 5 down | This protein is one of the nuclear-coded polypeptide chains of cytochrome c oxidase, the terminal oxidase in mitochondrial electron transport. |
| **MUC16** | Mucin-16 | 599 | 112 | 5 down | Thought to provide a protective, lubricating barrier against particles and infectious agents at mucosal surfaces. |

**Table S1.** *Cont.*

| **Gene Name** | **Protein Name** | **Normalized Signal** | | **Fold Change** | **FUNCTION** | |
| --- | --- | --- | --- | --- | --- | --- |
|  |  | **−3T3** | **+3T3** |  |  |  |
| **PSCA** | Prostate stem cell antigen | 103 | 20 | 5 down | May be involved in the regulation of cell proliferation. Has a cell-proliferation inhibition activity in vitro. | |
| **TJP3** | Tight junction protein ZO-3 | 248 | 48 | 5 down | Role in the linkage between the actin cytoskeleton and tight-junctions and also sequesters cyclin D1 at tight junctions during mitosis. | |
| **PLA2G10** | Group 10 secretory phospholipase A2 | 323 | 63 | 5 down | PA2 catalyzes the calcium-dependent hydrolysis of the 2-acyl groups in 3-sn-phosphoglycerides. Has a powerful otency for releasing arachidonic acid from cell membrane phospholipids. | |
| **ELF3** | ETS-related transcription factor  Elf-3 | 862 | 169 | 5 down | Transcriptional activator that binds and transactivates ETS sequences containing the consensus nucleotide core sequence GGA[AT]. Acts synergistically with POU2F3 to transactivate the SPRR2A promoter and with RUNX1 to transactivate the ANGPT1 promoter. Also transactivates collagenase, CCL20, CLND7, FLG, KRT8, NOS2, PTGS2, SPRR2B,TGFBR2 and TGM3 promoters. Represses KRT4 promoter activity. Involved in mediating vascular inflammation. May play an important role in epithelial cell differentiation and tumorigenesis. | |
| **DDIT3** | DNA  damage-inducible transcript 3 protein | 388 | 78 | 5 down | Member of the CCAAT/enhancer-binding protein (C/EBP) family of transcription factors, nhibits the DNA-binding activity of C/EBP and LAP by forming heterodimers that cannot bind DNA. | |
| **B3GALT4** | Beta-1,3-galactosyltransferase 4 | 77 | 15 | 5 down | Involved in GM1/GD1B/GA1 ganglioside biosynthesis. This gene is oriented telomere to centromere in close proximity to the ribosomal protein S18 gene. | |
| **CEACAM6** | Carcinoembryonic antigen-related cell adhesion molecule 6 | 7362 | 1478 | 5 down | One of the most widely used tumor markers in serum immunoassay determinations of carcinoma. Cluster of differentiation 66c. Cell surface glycoprotein that plays a role in cell adhesion and in intracellular signaling. | |
| **SPINK8** | Serine protease inhibitor Kazal-type 8 | 114 | 23 | 5 down | Probable serine protease inhibitor. |  |

**Table S1.** *Cont.*

| **Gene Name** | **Protein Name** | **Normalized Signal** | | **Fold Change** | **FUNCTION** |
| --- | --- | --- | --- | --- | --- |
|  |  | **−3T3** | **+3T3** |  |  |
| **HPGD** | 15-hydroxyprostaglandin dehydrogenase | 647 | 134 | 5 down | Prostaglandin inactivation. Contributes to the regulation of events that are under the control of prostaglandin levels. Catalyzes the NAD-dependent dehydrogenation of lipoxin A4 to form 15-oxo-lipoxin A4. Inhibits in vivo proliferation of colon cancer cells. |
| **P4HTM** | Transmembrane prolyl  4-hydroxylase | 173 | 36 | 5 down | Role in blood vessel development, adaptation to hypoxia and may be related to cellular oxygen sensing. Catalyzes the post-translational formation of 4-hydroxyproline in hypoxia-inducible factor (HIF) alpha proteins. |
| **STRA6** | Stimulated by retinoic acid gene 6 protein homolog | 70 | 15 | 5 down | Involved in the metabolism of retinol. This protein removes the retinol from the complex and transports it across the cell membrane. Defects in this gene are a cause of syndromic microphthalmia type 9 (MCOPS9). |
| **LIMCH1** | LIM and calponin homology domains-containing protein 1 | 99 | 21 | 5 down | Novel protein engaged in cell migration was identified in a systematic screening of misexpressing proteins in migratory border, associated with the sarcomeric type stress fiber. |
| **AQP9** | Aquaporin-9 | 212 | 1005 | 5 up | Water-selective membrane channels allows passage of a wide variety of noncharged solutes. It stimulates urea transport and osmotic water permeability; there arecontradicting reports about its role in providing glycerol permeability. |
| **KIFC1** | Kinesin-like protein KIFC1 | 467 | 223 | 5 up | Minus end-directed microtubule-dependent motor required for bipolar spindle formation. May contribute to movement of early endocytic vesicles. |
| **DLGAP5** | Disks  large-associated protein 5 | 401 | 199 | 5 up | Potential cell cycle regulator that may play a role in carcinogenesis of cancer cells. Mitotic phosphoprotein regulated by the ubiquitin-proteasome pathway. Key regulator of adherens junction integrity and differentiation that may be involved in CDH1-mediated adhesion and signaling in epithelial cells. |
| **TOP2A** | DNA topoisomerase  2-alpha | 65 | 319 | 5 up | Controls and alters the topologic states of DNA during transcription. Control of topological states of DNA by transient breakage and subsequent rejoining of DNA strands. Topoisomerase II makes double-strand breaks. |

**Table S1.** *Cont.*

| **Gene Name** | **Protein Name** | **Normalized Signal** | | **Fold Change** | **FUNCTION** |
| --- | --- | --- | --- | --- | --- |
|  |  | **−3T3** | **+3T3** |  |  |
| **CDKN3** | Cyclin-dependent kinase inhibitor 3 | 48 | 239 | 5 up | May play a role in cell cycle regulation. Dual specificity phosphatase active toward substrates containing either phosphotyrosine or phosphoserine residues. Dephosphorylates CDK2 at 'Thr-160' in a cyclin-dependent manner. This gene was reported to be deleted, mutated, or overexpressed in several kinds of cancers. |
| **SLC2A3** | Solute carrier family 2, facilitated glucose transporter member 3 | 132 | 678 | 5 up | Facilitative glucose transporter. Probably a neuronal glucose transporter. |
| **SALL2** | Sal-like protein 2 | 41 | 240 | 6 up | Probable transcription factor. Negative regulation of transcription from RNA polymerase II promoter. |
| **RENBP** | *N*-acylglucosamine 2-epimerase | 33 | 197 | 6 up | Catalyzes the interconversion of N-acetylglucosamine to N-acetylmannosamine. Binds to renin forming a protein complex called high molecular weight (HMW) renin and inhibits renin activity |
| **RASL11B** | Ras-like protein family member 11B | 30 | 187 | 6 up | RASL11B is a member of the small GTPase protein family with a high degree of similarity to RAS proteins. |
| **THRA** | Thyroid hormone receptor alpha, THRA protein | 17 | 106 | 6 up | Nuclear hormone receptor. High affinity receptor for triiodothyronine. Sequence-specific DNA binding transcription factor activity and negative regulation of DNA-dependent transcription. |
| **SYT11** | Synaptotagmin-11 | 58 | 379 | 7 up | Calcium sensors and mediate calcium-dependent regulation of membrane trafficking in synaptic transmission. The encode protein is also a substrate for ubiquitin-E3-ligase parkin. |
| **DSC1** | Desmocollin-1 | 268 | 1807 | 7 up | Component of intercellular desmosome junctions. Involved in the interaction of plaque proteins and intermediate filaments mediating cell-cell adhesion. May contribute to epidermal cell positioning (stratification) by mediating differential adhesiveness between cells that express different isoforms. Linked to the keratinization of epithelial tissues. |

**Table S1.** *Cont.*

| **Gene Name** | **Protein Name** | **Normalized Signal** | | **Fold Change** | **FUNCTION** |
| --- | --- | --- | --- | --- | --- |
|  |  | **−3T3** | **+3T3** |  |  |
| **MT4** | Metallothionein-4 | 15 | 105 | 7 up | Seems to bind zinc and copper. Could play a special role in regulating zinc metabolism during the differentiation of stratified epithelia. |
| **C5ORF13** | Neuronal protein 3.1 | 19 | 154 | 8 up | Roles in neural function. Ectopic expression augments motility of gliomas. Promotes also axonal regeneration. Functions in cellular differentiation. Induces differentiation of fibroblast into myofibroblast and myofibroblast ameboid migration. Increases retinoic-acid regulation of lipid-droplet biogenesisregulation of lipid-droplet biogenesis. |
| **CLIP3** | CAP-Gly  domain-containing linker protein 3 | 113 | 1114 | 10 up | Role in T cell apoptosis by facilitating the association of tubulin and the lipid raft ganglioside GD3. The encoded protein also functions as a scaffold protein mediating membrane localization of phosphorylated protein kinase B. |
| **LOX** | Protein-lysine  6-oxidase | 44 | 576 | 13 up | Responsible for the post-translational oxidative deamination of peptidyl lysine residues in precursors to fibrous collagen and elastin. In addition to cross-linking of extracellular matrix proteins, may have a direct role in tumor suppression. |
| **OSR1** | Protein odd-skipped-related 1 | 35 | 632 | 18 up | Transcription factor that plays a role in the regulation of embryonic heart and urogenital development. |

© 2013 by the authors; licensee MDPI, Basel, Switzerland. This article is an open access article distributed under the terms and conditions of the Creative Commons Attribution license (http://creativecommons.org/licenses/by/3.0/).
